# Strychnia in Snakebite

**Published:** 1889-04-13

**Authors:** 


### Strychnia in Snakebite.

Although poisonous snakes are not among the plagues of our country, there is an interest felt in them and their doings that can only be accounted for by our having inherited an antipathy to them from our first parents, and the public in general feels a satisfaction at learning of a cure for snakebite that it does not experience on hearing of a remedy for diseases relatively more common.

Hitherto all antidotes to snake poison have been administered on the theory that the virus affected the blood, and the most successful results have come from the administration of alcohol, which seemed to maintain the strength of the sufferer till the poison was eliminated by natural means. This implies, of course, that only the milder cases could be cured, for the strength given by alcohol is of the most fleeting kind. An Australian doctor, however, Dr. Mueller, of Yackandandab, has recently come to the conclusion that the poison in snakebite affects the nerves, weakening and paralysing them, and has given a fair proof of the accuracy of his hypothesis by successfully treating a number of cases by one of our strongest nerve tonics, strychnia. In fact, it seems that snake venom is not, in the common sense, a poison at all; it does not directly cause any change of tissue. Its effect, therefore, is simply produced by the operation of dynamic force; it suspends the action of the nerve cells for a longer or shorter period.

Yet in death from snakebite the blood is found greatly changed. Dr. Mueller's explanation of this is that the pulmonary capillaries, through which the blood corpuscles pass, when going to the lungs to exchange the carbonic acid of effete blood for fresh and life-giving oxygen, have lost their power. They owe their tension—their healthy contracting power—to the influence of the vaso-motor nerves, and when the latter are paralysed, the capillaries lose their power of, so to speak, squeezing out the superfluous carbonic acid, and leaving the corpuscles free to take up oxygen. Thus the corpuscles pass through the lungs unchanged, carrying back to the heart blood as full of carbonic acid as they brought from it, and they themselves absolutely die, bursting in consequence of this load of carbonic acid.

This is Dr. Mueller's theory of the blood changes due to snakebite, a theory interesting chiefly to pathologists, while the knowledge of a certain, or even tolerably probable, cure for snake poisoning interests the whole community. And this seems to have been found in strychnia. The best direct preventatives of the fatal action of snake venom hitherto employed have been the excision of the part bitten and ligature above the part, both intended to prevent the poison circulating through the system, both efficient if the operation could be thoroughly done, but from the difficulty of effecting this, both unreliable. Alcohol, as already stated, is only an indirect cure, and the method of inoculation with the venom of another snake or reptile, as practised in South Africa, mentioned in our columns a few weeks ago, is not always possible. In this connection it would be interesting to know assuredly if the result of successful inoculation is to set up a rival dynamic force, which "fights it out" with that caused by the original poison to a Kilkenny cat termination.

If the injection of from 10 to 20 minims of liquor strychnia is really a cure for snake poisoning, Dr. Mueller deserves the thanks of the civilised world for his discovery. The symptoms of snake poison are coma, more or less complete, with dilation of the pupils, and a weak and irregular pulse. Dr. Mueller injects the strychnia by means of the hypodermic syringe, applies artificial heat, and strenuously interferes with the patient's desire for sleep. One curious circumstance he notes—that where alcohol has been administered before the injection of strychnia, one of the first symptoms of returning life is the development in the patient of the ordinary evidences of intoxication.

